# HIV-1 infection increases microRNAs that inhibit Dicer1, HRB and HIV-EP2, thereby reducing viral replication

**DOI:** 10.1371/journal.pone.0211111

**Published:** 2019-01-25

**Authors:** Shira Modai, Luba Farberov, Eytan Herzig, Ofer Isakov, Amnon Hizi, Noam Shomron

**Affiliations:** 1 Faculty of Medicine, Tel Aviv University, Tel Aviv, Israel; 2 Gladstone Institute of Virology and Immunology, University of California San Francisco, San Francisco, CA, United States of America; Universitat des Saarlandes, GERMANY

## Abstract

HIV-1 is the causative agent of AIDS (Autoimmune Deficiency Syndrome). HIV-1 infection results in systemic CD4^+^ T cell depletion, thereby impairing cell-mediated immunity. MicroRNAs are short (~22 nucleotides long), endogenous single-stranded RNA molecules that regulate gene expression by binding to the 3' untranslated regions (3' UTR) of mRNA transcripts. The relation between HIV-1 infection and human miRNA expression profile has been previously investigated, and studies have shown that the virus can alter miRNA expression and *vice versa*. Here, we broaden the understanding of the HIV-1 infection process, and show that miRNA-186, 210 and 222 are up-regulated following HIV-1 infection of human Sup-T1 cells. As a result, the host miRNA target genes: Dicer1 (Double-Stranded RNA-Specific Endoribonuclease), HRB (HIV-1 Rev-binding protein) and HIV-EP2 (Human Immunodeficiency Virus Type I Enhancer Binding Protein 2), are down-regulated. Moreover, testing the miRNA-gene anti- correlation on the Jurkat and the HeLa-MAGI cell lines demonstrated the ability of the miRNAs to down-regulate viral expression as well. To conclude, we found that human miR-186, 210 and 222 directly regulate the human genes Dicer1, HRB and HIV-EP2, thus may be filling key roles during HIV-1 replication and miRNA biogenesis. This finding may contribute to the development of new therapeutic strategies.

## Background

Infection with the human immunodeficiency virus type 1 (HIV-1) results in systemic CD4 T cell depletion that impairs cell-mediated immunity; this leads to numerous opportunistic infections and cancers. Therefore, the overall medical condition caused by HIV-1 infections is called Acquired Immunodeficiency Syndrome (AIDS). Rates of both infection and death relating to HIV-1 have been declining, due to lifelong treatment by a cocktail of 3–4 medications. Nonetheless, about 36.7 million people are currently living with HIV-1 and approximately 1 million die from it annually (UNAIDS Data, 2017). Since 1981, when the first cases of HIV-1 infections were reported, detection, control, and the eventual elimination of the viral HIV-1 infection and AIDS have become worldwide goals. Achieving these goals has proven to be highly challenging, as HIV-1 has become increasingly resistant to therapy, by evading the immune response, as well as by altering cellular immune function and protecting infected cells from apoptosis[1–3]. The combination of Anti-retroviral Therapy (cART), which is used to suppress HIV-1 to undetectable levels in patients' plasma, is a major medical success. Nevertheless, HIV-1 is far from being completely eliminated, and the development of novel means to completely suppress viral replication is of high importance.

The HIV-1 life cycle involves numerous competing and complementary interactions of viral RNAs with viral and cellular proteins, some of which can inhibit the infection, thus providing an opportunity for therapeutic interventions[4–7]. Virus entry, for instance, includes the host receptors CD4 with co-receptors CCR5 or CXCR4 to promote host and viral membranes fusion. Uncoating of the viral membrane, entry of the capsids and assembly of the Pre-integration complex (PIC) includes the viral DNA and proteins (such as Vpr (viral protein R), matrix and integrase), but also host proteins like BANF1 (Barrier to autointegration factor 1). After integration is done, the HIV-1 transactivator protein (Tat) stimulates transcription from the HIV-1 long terminal repeat (LTR) by interacting with the viral transactivation response (TAR) element and recruiting another group of host proteins called P-TEF-b (Positive Transcription Elongation Factor b), which stimulate transcriptional elongation. The HIV master regulator Nef forces an environment suitable for dynamic viral production via up regulation of YY1 (Yin-Yang 1) which is an HIV negative regulator but also HIV-EP2 (HIV enhancer binding protein 2) which activates T-cell for productive replication. The release of incompletely spliced HIV-1 RNAs from the perinuclear region to the cytoplasm is promoted by the viral Rev protein. It binds HIV mRNA that contains the RRE (REV Responsive Element) sequence with the support of the host proteins HRB (HIV Rev Binding protein), CRM1 (chromosome region maintenance 1) and RanGTP. Additional interactions between the HIV-1 and the cell are known, but not to a full extent, and others are yet to be revealed.

MicroRNAs (miRNAs) are short (~22 nucleotides long), endogenous single-stranded RNA molecules that regulate gene expression by binding to the 3' untranslated regions (3' UTR) of mRNA transcripts[8–10]. miRNAs are key regulators in diverse biological processes, such as development, differentiation, proliferation, growth, cell cycle, apoptosis and stress response[11]. miRNA biogenesis starts with an initial, primary transcript (pri-miRNA), produced by the RNA polymerase II enzyme. This RNA sequence contains a stem-and-loop secondary structure that undergoes nuclear cleavage by the Microprocessor complex. The pri-miRNA cleavage product is a 70-110bp stem-loop intermediate, known as the precursor miRNA (pre-miRNA)[12]. After export to the cytoplasm, the pre-miRNA undergoes a subsequent cleavage by the RNase III Dicer (transcribed from the Dicer1 gene) to a ~22bp duplex. One strand then incorporates with Argonaute (Ago2) proteins into the RNA Inducing Silencing Complex (RISC)[13], which enables recognition and suppression of the matching mRNA target.

The relation between HIV-1 infection and miRNA biogenesis is well documented. Over 1000 miRNAs are encoded in the human genome, and each one is able to target multiple mRNAs. It seems that there are numerous possibilities in which cellular proteins could be involved in HIV replication. Previous studies have described the ability of HIV-1 infection to alter miRNA profiles on one hand; and the effects of miRNAs on HIV-1 pathogenesis, on the other (reviewed in[14]). Comparing HIV-1-infected versus non-infected cells by microarray analysis, it was shown that some cellular miRNAs were up-regulated, whereas others were down-regulated by HIV-1. These findings argue for an alternative mechanism to explain how specific genes and miRNAs are modulated by HIV-1. In one case cellular miR-29a was shown in HIV-1-infected human T lymphocytes to target HIV-1 3’UTR region and inhibit HIV-1 production and infectivity. In other cases, the cellular miRNA expression profile of SUP-T1 cells at earlier time points (5, 12, and 24 h post-infection) has demonstrated a phased pattern of miRNA expression, wherein many miRNAs that were suppressed during early time points post virus challenge were then upregulated at later time points post infection.

We recently conducted a detailed analysis of miRNA expression in cells that were infected by HIV-1, and identified 151 miRNAs that were differentially expressed; some of them enabled a more efficient infection process[15]. In this study, we hypothesize that there is a group of human miRNAs which are involved in HIV-1 replication that may act to eliminate the infection. We continued our analysis of miRNA expression in the HIV-1 infected human Sup-T1 cell line by focusing on miR-186, miR-210 and miR-222, and by showing their ability to inhibit viral replication via the down-regulation of the host mRNA molecules of Dicer1, HRB (HIV-1 Rev-binding protein) and HIV-EP2 (Human Immunodeficiency Virus Type I Enhancer Binding Protein 2).

## Materials and methods

### Cell culture

Suspension cells Sup-T1 (Human T-cell lymphoblastic lymphoma) and Jurkat (Human acute T-cell leukemia cells), were cultured in RPMI medium 1640, supplemented with 10% (vol/vol) heat-inactivated fetal bovine serum (FCS), 0.3 g/liter L-glutamine, 100 units/ml penicillin, and 100 units/ml streptomycin (Biological Industries, Kibbutz Beit Haemek, Israel).

Monolayer-adherent MCF7 (human adenocarcinoma breast cell line) and HeLa MAGI-CCR5 (HeLa-CD4-LTR-β-gal)[16,17], which are HeLa derived cells that express high levels of CD4 and CCR5, and a single integrated copy of a β-galactosidase under a truncated HIV-1 LTR control to indicate HIV-1 infection, were grown in Dulbecco's Modified Eagle Medium (DMEM) supplemented with 10% (vol/vol) FCS, 0.3 g/liter L-glutamine, 100 units/ml penicillin, and 100 units/ml streptomycin (Biological Industries, Kibbutz Beit Haemek, Israel).

MCF7 cells were supplied by Prof. Ilan Tsarfaty, Tel-Aviv University.

HeLa MAGI-CCR5 cells were supplied by the AIDS-reagent program. Sup-T1 cells were supplied by Prof. Dan Peer, Tel-Aviv University. Jurkat cells were supplied by Prof. Ronit Sagie-Eisenberg, Tel-Aviv University.

All cells were incubated at 37oC in 5% CO_2_. Cells were counted with 1:1 trypan blue 0.5% (Biological Industries, Kibbutz Beit Haemek, Israel) using Countess, an automated cell counter (Life Technologies, USA) prior to each experiment.

### HIV-1 preparation

HIV-1_HXB2_ –HIV-1 from HXB2 isolate was produced using the pSVC21 plasmid (full length HIV-1_HXB2_ strain) as previously described[18]. Briefly, HIV-1 (pSVC21) wild type plasmid was used to transfect HEK293 cells with the TurboFect Cell Transfection Reagent (Fermentas, Lithuania), according to the manufacturer's instructions. To identify virus titters, the transfected cells were cultured in DMEM complete medium for 24, 48 or 72 hours. At each time point, the medium was removed and filtered through a 0.45μM filter (Millipore, USA). The number of virions in the medium was analyzed with RT-qPCR (see below) using virus specific U5 primers. All the following experiments were based on the virus titer generated in the 48 hours post infection time point.

### Viral infection assays for next generation sequencing

*HIV-1 Sup-T1 infection*: Two million Sup-T1 lymphocyte cells were infected with 4 x10^4^ HIV-1_HXB2_ virions, MOI = 0.02. Four days later, 2 million fresh cells were added to the culture. To achieve a high infection rate[15,19], eight days post-infection (PI), cells were harvested and total RNA was extracted using TRIzol according to the manufacturer’s instructions. RNA was analyzed using next generation sequencing, TaqMan Low-Density Arrays (TLDAs), and RT-qPCR (see detailed description below).

### Viral replication assays

MAGI-CCR5 cells were seeded in 6-well plates at a concentration of 2 x10^5^ cells/well. miRNAs were transfected using Lipofectamine 2000 (Invitrogen, Carlsbad, CA, USA) according to the manufacturer’s instructions, with one of five plasmids: miR-Vec-186, miR-Vec-210, miR-Vec-222, empty-miR-Vec and pEGFP, in triplicates. Twenty-four hours after transfection, the cells were counted and seeded in a 96-well plate (Greiner Bio-One, Austria) at a concentration of 1x10^4^ cells/well. Six hours later, when cells reached 30% confluency, the culture medium was removed and each well was infected with 50μl HIV-1 (HXB2 strain diluted to MOI of 0.01 with complete DMEM in the presence of 20μg/ml DEAE-Dextran). Two hours PI, 150μl of complete DMEM was added to each well. Twenty-four hours PI, supernatant was collected from each well for RNA extraction and RT-qPCR was conducted to assess viral load. Five microliters of MMLV were added to the supernatant and served as a control for RNA extraction, Viral RT-qPCR

HIV-1 quantification primers[15]:

Forward–TGGTAATAACAACATATTGGGGT

Reverse–CCTGACCCAAATGCCAGT

MLV control quantification primers[20]

Forward–CTCTAATGGCCACTCAGCAA

Reverse–CCTCCCTGAGATCATCCTGT

### Plasmid cloning

All inserts were PCR amplified from Jurkat genomic DNA (for miRNA constructs and 3'UTR constructs) using Phusion Flash High-Fidelity DNA Polymerase (Thermo Fisher Scientific, USA) under the following conditions: 98°C for 10 seconds, 30 cycles of: 98°C for 10 seconds, 60°C for 5 seconds, 72°C for 15 minutes, and final extension at 72°C for 10 minutes. Double digestion was performed in the presence of two restriction enzymes (New England Biolabs (NEB), USA) and their compatible buffer for 1 hour at 37°C, according to the manufacturer’s instructions. The restriction product was run on a 1.5% agarose gel, excised and then purified using Wizard SV Gel and PCR Clean-Up kit (Promega, USA) to eliminate excess nucleotides and primers. The clean product was then ligated to the complementary plasmid using T4 DNA ligase (NEB, USA) for 1 hour at 37°C and transformed into DH5α competent *E*. *coli* cells (Bio-Lab, Israel). Cells were grown overnight on 0.1% ampicillin LB-agar plates at 37°C. Colonies were selected and grown overnight in a 37°C shaker in 0.1% ampicillin liquid LB. Plasmids were extracted using the NucleoSpin midi-prep kit (Macherey-Nagel, Germany) and were Sanger-sequenced for verification.

### miRNAs constructs

The genomic regions of the human pre-miRNA-186, 210 and 222 were cloned into separate retroviral vectors, in a *Bam*HI-*Eco*RI restriction site under a strong CMV promoter, called miRVec[21] (Professor Reuven Agami, The Netherlands Cancer Institute). miRVec-186 and miRVec-222 were received from Prof. Agami's lab. miRVec-210 was prepared with the genomic loci of ~70 bp, upstream and downstream of the pre-miRNA, by PCR-amplification from the Jurkat cell line genomic DNA. *Bam*HI–*Eco*RI restriction sites (as indicated by bold letters) were added to the PCR primers:

miRVec-210 Forward: gc**g**g**atcc**tcggacgcccaagttggagg

miRVec-210 Reverse: gc**gaattc**tgccctcgcgtccccgtgtg

### 3'UTR constructs

Fragments of the 3' UTR of HIV-EP2, HRB and Dicer1 mRNA, spanning the matching miR-186/210/222 binding sites, were cloned into the *Xho*I–*Not*I restriction site, downstream of the Renilla luciferase reporter of the psi-CHECK-2 plasmid, under the T7 promoter. This plasmid contains a firefly luciferase reporter, which is used as a control under the HSV-TK promoter (Promega, USA). The 3' UTR fragments were PCR amplified as described above. *Xho*I–*Not*I restriction sites were added to the following primers (restriction sites for *Xho*I forward primers and *Not*I reverse primers are marked in bold letters; forward primers are indicated by “F,” and reverse primers by “R”) (see S1 Table).

Four-nucleotide mutations in the seed binding region of these cloned 3'UTRs were generated using PCR site-directed mutagenesis (SDM), according to the overlap expansion protocol[22] (see primers in Table T1, and the sequences of the WT and mutated 3'UTR against the miRNA seed).

The SDM reaction was performed in the following conditions: 95oC for 2 minutes; 18 cycles of: 95oC for 20seconds, 60oC for 10 seconds and 68oC for 2 minutes; and finally, 68oC for 5 minutes. Products were treated with *Dpn*I (NEB, USA) to digest the original methylated plasmid. The new vectors were sequenced for verification.

### RNA extraction

Total RNA was extracted using TRIzol reagent (Invitrogen, Life Technologies) according to the manufacturer's instructions. RNA concentration and purity were measured using a NanoDrop ND-1000 spectrophotometer (NanoDrop Technologies, Thermo Scientific, USA). The samples that were analyzed by next generation sequencing underwent additional quality control using gel electrophoresis. RNA was separated in Tris-Acetate-EDTA (TAE) on a 1.5% agarose gel supplemented with ethidium bromide for 60 minutes at 100 volts and then visualized and photographed under UV light.

### Small RNA next generation sequencing library preparation

Library preparation was conducted with 10 μg of each sample following Illumina’s TruSeq Small RNA sample preparation protocol (v1.5). During this process, samples were ligated with 3′ and 5′ adapters, reverse-transcribed and then PCR amplified. Libraries of cDNA were prepared from ∼100 bp PCR products (representing ∼25 nt RNA molecules) and sequenced each on a separate lane of an Illumina Genome Analyzer IIx at the Tel Aviv University Genome High-Throughput Sequencing Laboratory.

### Illumina next generation sequencing data analysis

The data sequences were screened for the sequence of the small RNA adapter, and the adapter sequences were trimmed using standard settings in Illumina’s GAPipeline1.0.

Illumina data were processed in RandA software[23]. Reads were aligned to the human subset miRNAs in the miRbase database (http://www.mirbase.org/)[24] using BWA-aligner software (http://bio-bwa.sourceforge.net/index.shtml)[25]. The number of reads was standardized by mapping each transcript according to its length; and the initial total number of mapped reads in the sample was based on the "reads per kilo-base per million" (RPKM) method[26]. Only perfect matches were counted in the main analysis. Next, results were ranked in terms of miRNAs that were differentially expressed (S2 Table), comparing the infected sample to the uninfected one. Statistical analysis was performed using a chi-square distributed statistic that showed the differentiability in expression, while accounting for the differences in size (number of mapped reads) of both lanes and samples.

Additional processing by genome matching was assessed with ELAND software (Illumina) using the *Homo sapiens* full genome hg19 (available from UCSC[27]) and Human Immunodeficiency Virus 1, complete genome (HXB2 strain—NC_001802.1 GI:9629357). Additionally, the miRNAkey software pipeline was used for the analysis of microRNA next generation sequencing data[28].

### miRNA profiling arrays

One microgram of total RNA was used to generate cDNA using the TLDAs, which are RT–qPCR assays based on specific stem–loop primers, each of which is complementary to a specific mature miRNA. These primers are provided in a mixture that generates a multiplex PCR reaction (megaplex). Procedures were profiled and analyzed as previously described[29]. Briefly, first-strand cDNA was synthesized using the High Capacity cDNA kit (Life Technologies). Complementary DNA (cDNA), RNase-free water, and TaqMan Universal PCR Master Mix No AmpErase UNG (Life Technologies) were then infused into a loading port on Human TLDA card A (v2.0), centrifuged twice and sealed according to the manufacturer’s instructions. PCR amplification was performed on an ABI Prism 7900HT Sequence Detection System under the following conditions: 2 minutes at 50oC, 10 minutes at 95oC and 40 cycles: 30 seconds at 95oC and 1 minute at 60oC. Results were analyzed with SDS software (Applied Biosystems / Life Technologies). Relative levels of miRNA were calculated based on the comparative threshold cycle (Ct) method. -ΔΔCt = -[(normalized number of reads in the HIV-infected sample)–(normalized number of reads in the non-infected sample)]. Fold Change = log2(-ΔΔCt). U6 snRNA was used for normalization, which remained stable under the different treatments. Reactions were run on an Applied Biosystems 7900HT Fast Real-Time PCR System (Life Technologies).

### RT-qPCR assays

Reverse transcription of mRNA was achieved using the High-Capacity cDNA Reverse Transcription Kit with random primers. Reverse transcription reactions for specific mature miRNAs were performed using TaqMan miRNA Assays, according to the manufacturer’s protocol (Life Technologies). Single miRNA/mRNA expression was similarly assessed using TaqMan Universal PCR Master Mix No AmpErase UNG (Life Technologies) or SYBR green Fast PCR master mix (Life Technologies). PCR amplification and reading of mRNA and miRNA expression were performed in triplicate using the Step-One Sequence Detection System (ABI, Life Technologies).

mRNA expression was quantified under the following thermal cycler conditions: 20 seconds at 95°C, 40 amplification cycles of: 3 seconds at 95°C and 30 seconds at 60°C; and a melt curve: 15 seconds at 95°C, 1 minute at 60°C and 15 seconds at 95°C. Expression levels were calculated based on the Ct method. mRNA expression levels were normalized to glyceraldehyde 3-phosphate dehydrogenase (GAPDH) as an endogenous control.

Mature mRNA expression was quantified under the following thermal cycler conditions: 2 minutes at 50°C, 10 minutes at 95°C and 40 amplification cycles (15 seconds at 95°C and 1 minute at 60°C). miRNA levels were normalized to U6 and are shown as fold change relative to controls. Gene qPCR primers:

GAPDH Forward: CAAGAAGGTGGTGAAGCAGG

GAPDH Reverse: GGCCATGAGGTCCACCAC

Dicer1 Forward: ATTTTGCACTTACCCTGATGC

Dicer1 Reverse: CAGGGGGATCAAATATTGACA

HIV-EP2 Forward: TCTTCTGAGGTCCAAGCAAAA

HIV-EP2 Reverse: GGACGCATCAGGTTTCATCT

HRB Forward: CAAGAAAAGTATGAAAAGAAAAGATGG

HRB Reverse: CCTCAGGTGTGCTGCTTGT

## Bioinformatics analysis

TargetRank (http://genes.mit.edu/targetrank/)[30] and TargetScan 5.1 (http://www.targetscan.org/)[31] were used to predict target genes of the examined miRNAs. The miRBase (http://www.mirbase.org/)[24] and UCSC genome browser (https://genome.ucsc.edu/)[27] were used to obtain sequences of the miRNAs and their predicted 3'UTR target genes, as well as for *in silico* PCR prior to ordering primers. RNAfold webserver (http://rna.tbi.univie.ac.at/cgi-bin/RNAWebSuite/RNAfold.cgi) was used to predict secondary structures and the amount of free energy released when the 3'UTR of the target genes are spontaneously folded.

### miRNA transfections

Transfections for non-infection assays were carried out 24 hours after seeding cells. All over-expression assays were conducted with Lipofectamine 2000 (Invitrogen, USA) according to the manufacturer’s instructions. Transfection efficiency was measured using Enhanced Green Fluorescent Protein (EGFP), expressed from 5ng of pEGFP that was transfected in a separate well. Only wells with transfection efficiency greater than 80% were used in downstream applications.

### miRNA over-expression

HeLa-CCR5 cells were seeded in six-well plates at a concentration of 2 ×10^5^ cells/well. Cells were transfected with 1.25ug of miRVec plasmids (miR-186, miR-210, miR-222 or control empty plasmid). Twenty-four hours later, cells were either used for RNA purification or infected with HIV-1.

### Luciferase assays

MCF7 cells were seeded in 96-well plates at a concentration of 8 x 10^3^ cells/well. Each well was transfected with one of four combinations of plasmids: 1ng of the 3'UTR binding site, with or without its suspected miRNA (97ng); or the mutated 3'UTR binding site, with or without its suspected miRNA. Twenty-four hours later firefly and Renilla luciferase activities were measured using the Dual-Luciferase Reporter Assay kit (Promega, USA). The LUMIstar Omega Luminometer (BMG LabTech, Germany) was used to read the intensities. The Renilla luciferase results were normalized to the values of the firefly luciferase.

### Statistical analysis

Data were expressed as mean ± standard deviation (SD) from three independent experiments. The significance of differences between groups was determined by the Student’s *t-*test in all experiments. Values of *P* < 0.05 were considered statistically significant.

## Results

### HIV-1 infection induces a shift in human miRNA expression profiles

**Fig 1:**
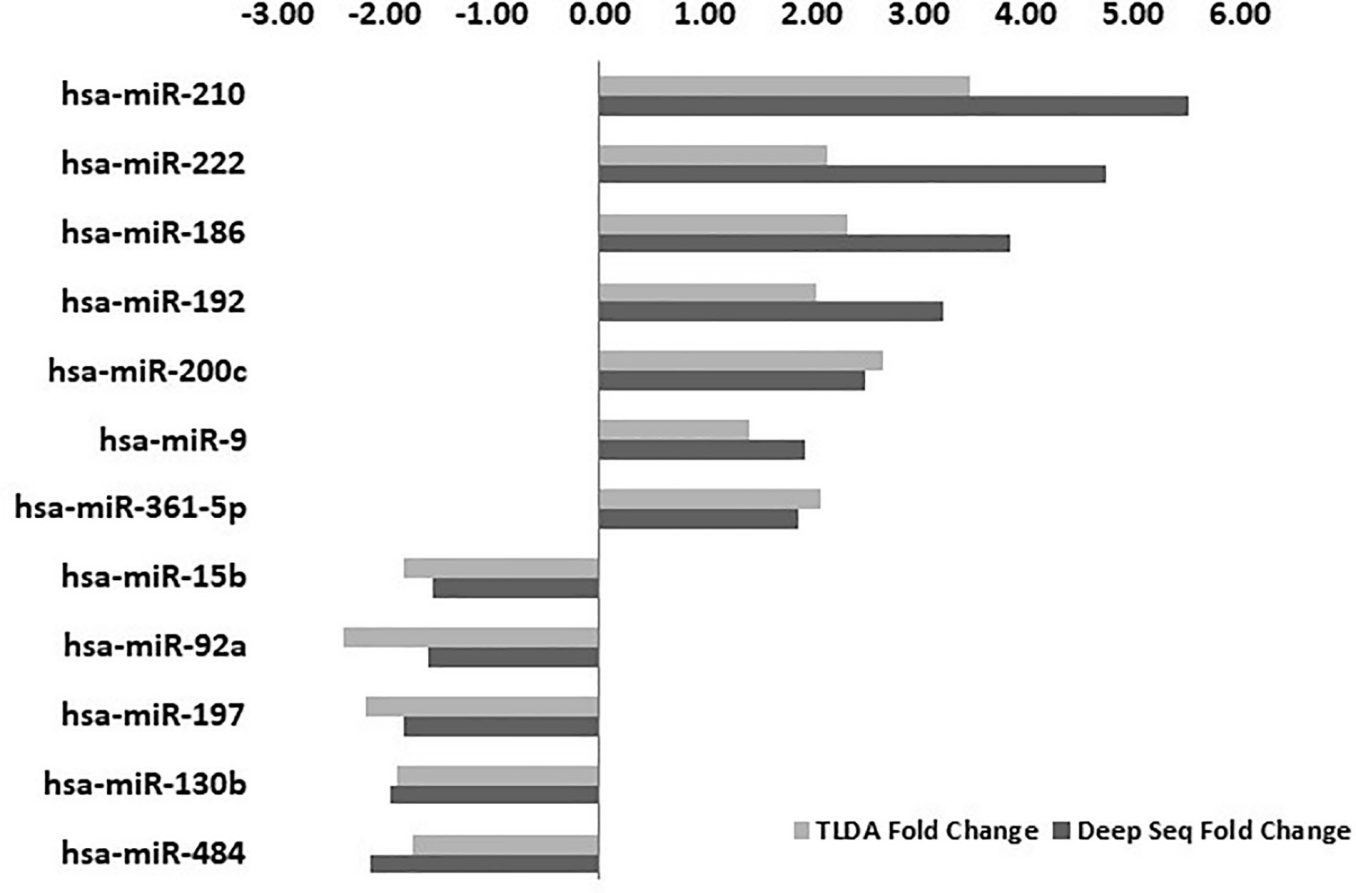
miRNA expression levels following HIV-1 infection. Two methods were used to profile miRNA levels: NGS (dark grey bars) and TaqMan Low Density Array (TLDA) (light grey bars). NGS data were normalized using the "reads per kilo-base per million" method. -ΔΔCt = -[(normalized number of reads in the HIV-infected sample)–(normalized number of reads in the non-infected sample)]. TLDA data were normalized, using U6 snRNA, and -ΔΔCt was calculated using the ΔΔCt method (for details see the Materials and Methods).

The reciprocal interactions between micro-RNAs and HIV pathogenesis is a growing field of research. To better understand the miRNA regulation process during HIV-1 infection, we used NGS to profile the small non-coding RNAs (ncRNAs) of HIV-1-infected and non-infected Sup-T1 cells. Sequencing reads were aligned to the human and HIV-1 genomes. The miRNA profiles were validated using an RT-qPCR-based method, known as TLDA (TaqMan Low Density Arrays). The two datasets were compared and 12 miRNAs with the highest correlations between both methods were further analyzed (Fig 1).

### miRNAs-186, 210 and 222 down-regulate HRB, HIV-EP2 and Dicer1 mRNAs

**Fig 2:**
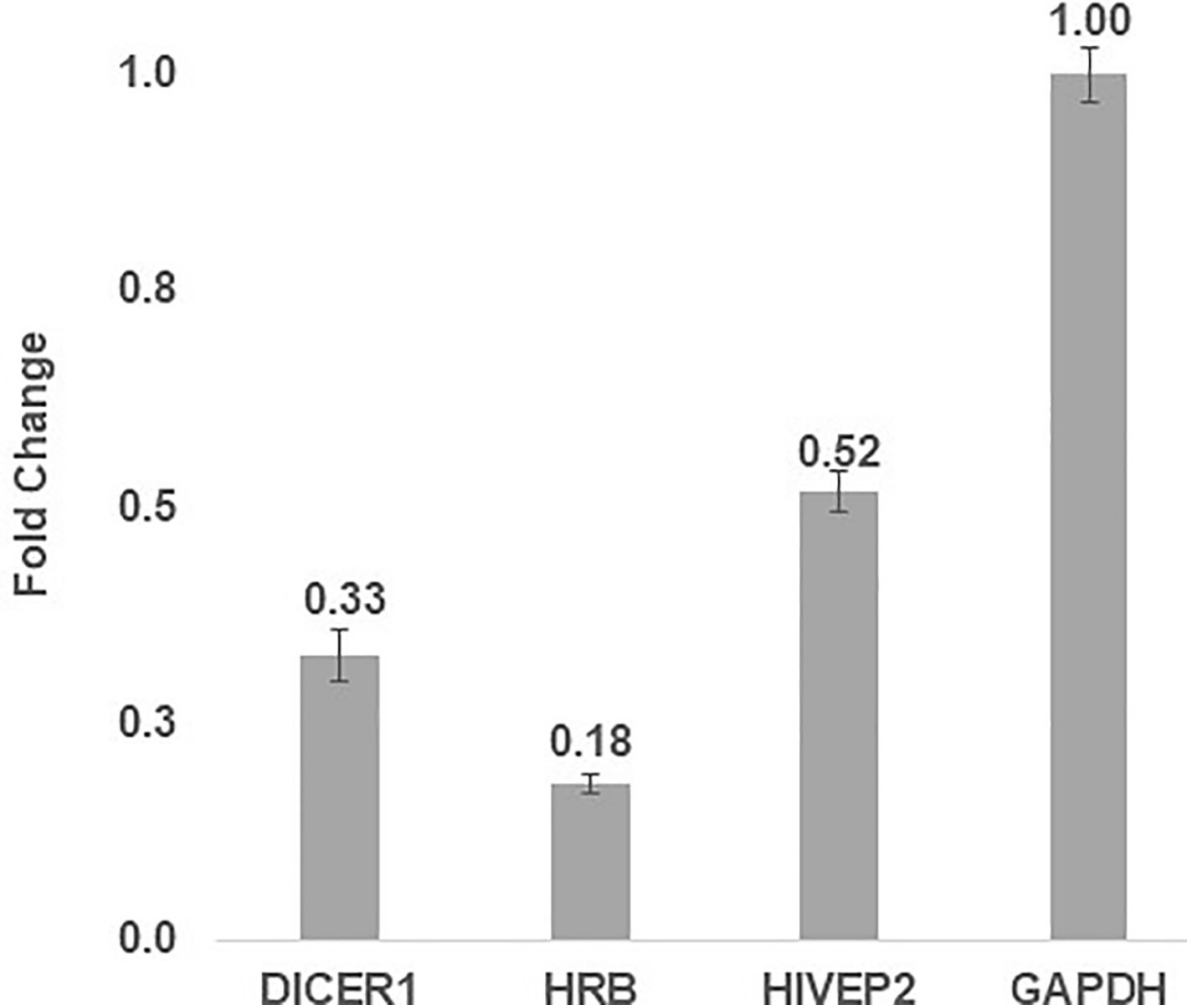
Overlapping mRNA targets of miRNA-186, 210 and 222 are decreased during HIV-1 infection. Expression fold change values of predicted mRNA targets in HIV-1 infected Sup-T1 cells, compared to uninfected Sup-T1 cells, using RT-qPCR normalizer GAPDH (3 technical repetitions).

miR-186, 210 and 222 were the miRNAs that were most up-regulated following HIV-1 infection (as measured by both methods), and thus were selected for further experiments. To understand the implications of this up-regulation, we analyzed their target genes, using algorithms that seek conserved complementary binding sites on each gene’s 3’UTR (TargetScanHuman 5.1. at http://www.targetscan.org/). We crossed their list of potential targets and searched for conserved miRNA binding sites that were putative targets of at least two of the miRNAs tested, and that are known to be components of the viral replication cycle. The genes that were identified by this analysis were Dicer1, HRB and HIV-EP2 (see the level of conservation of the miRNAs families and the seed, and the number of miRNA binding sites in the 3'UTR). Indeed, mRNA levels of these genes in the same experimental samples were decreased (48% and up to 82%, Fig 2), as expected, given the increase of their regulating miRNAs.

**Fig 3:**
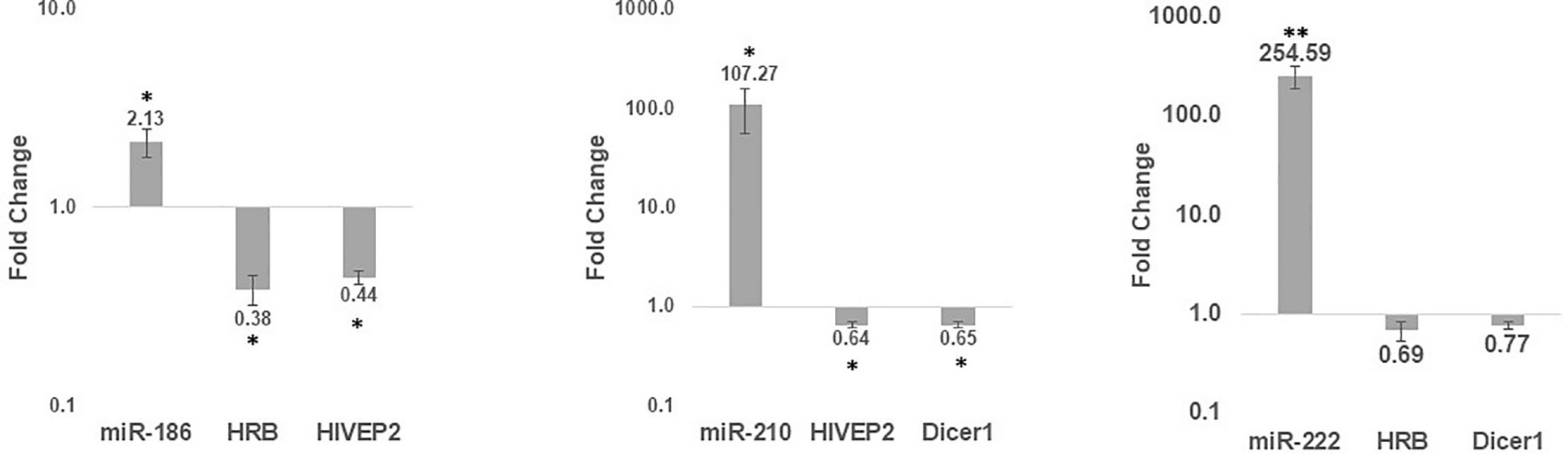
miRNAs-186, 210 and 222 promote down-regulation of HRB, Dicer1 and HIV-EP2 mRNA expression. miRNAs miR-186, 210 and 222 were over–expressed in the Jurkat cell line (in triplicates). After 24 hours, RNA was extracted, and cDNA was prepared and assayed by RT-qPCR of miRNAs and their predicted targets. U6 snRNA and GAPDH were used for the background of miRNA and mRNA, respectively. Fold change was calculated compared to a control plasmid, using the ΔΔCt method. The paired Student t-test was used for statistical analysis (n = 3, * p<0.05, ** p-value<0.005).

To corroborate our results, we examined the reciprocal miRNA-mRNA interaction. We over-expressed miR-186, 210 and 222 in the Jurkat cell-line, using a miRNA vector. Following 24 hours of miRNA over-expression, target gene levels were reduced by 23% to 62%, as depicted in Fig 3. An attempt to perform an antagomir experiment to reduce the miRNAs and consequently increase its target genes was unsuccessful (data not shown).

**Fig 4:**
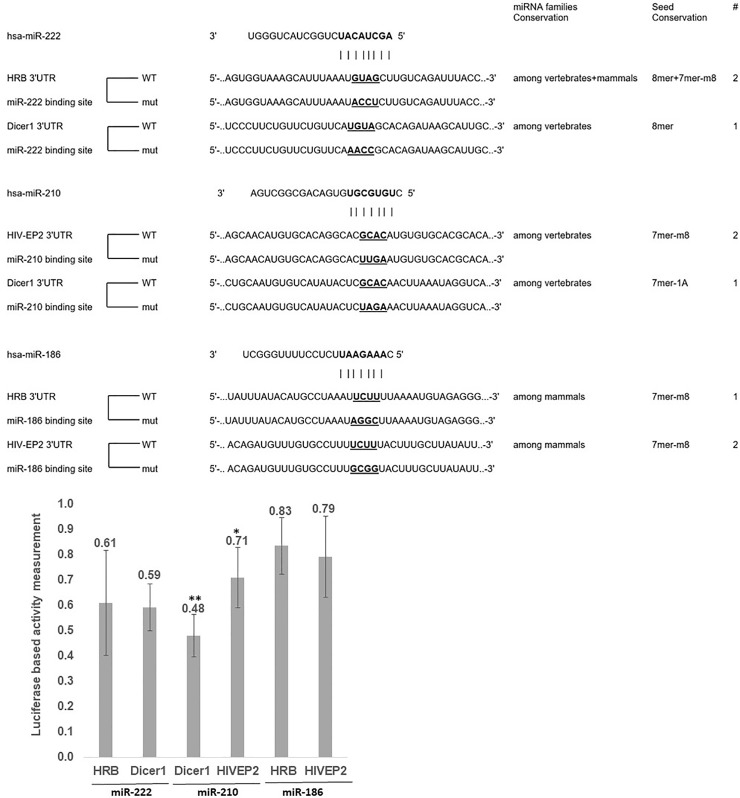
miRNA-186, 210 and 222 directly regulate Dicer1, HRB and HIV-EP2. (A) The sequences of Renilla and firefly luciferase under regulation of Dicer1, HRB and HIVEP2 3′UTRs, which were used for transient reporter assay experiments. Each of the three miRNA-binding sites is presented with its wild-type (WT) and mutant (mut) alleles. The miRNA seed region is marked in bold, and the mutated nucleotides in the complimentary 3′UTR sequence are marked in bold and underlined. The number of binding sites and conservation of the miRNAs are detailed. The level of conservation of the miRNAs families and the seed and the number of miRNA binding sites in the 3'UTR are decribed on the right. (B) miRNA-186, 210 or 222 were over–expressed in the MCF7 cell line in combination with either of the 3′-UTR constructs (as described in the Materials and Methods). Twenty-four hours post- transfection, the cells were lysed and luminescence was measured. Reduction in luminescence was calculated as follows: (3'UTR+miRNA/3'UTR-miRNA) / (mutated 3'UTR+miRNA/mutated 3'UTR-miRNA). The paired Student t-test was used for statistical analysis (n = 3, * p<0.05, ** p <0.005).

Then, we examined the direct regulation of the different putative targets by each miRNA, using the dual luciferase reporter assay (Fig 4A). We measured the luminescence generated in the reaction (S2 Table), from which we calculated the level of direct binding between every miRNA and its target gene. Our results demonstrate the down-regulation of all three genes: Dicer1 by miR-210 and by miR-222 (52% and 41%, respectively); HRB by miR-222 and by miR-186 (39% and 17%, respectively); and HIV-EP2 by miR-210 and by miR-186 (29% and 21%, respectively) (Fig 4B). Therefore, we established a direct interaction of binding of the miRNAs with those genes.

### miRNA-186, 210 and 222 are involved in HIV-1 replication

**Fig 5:**
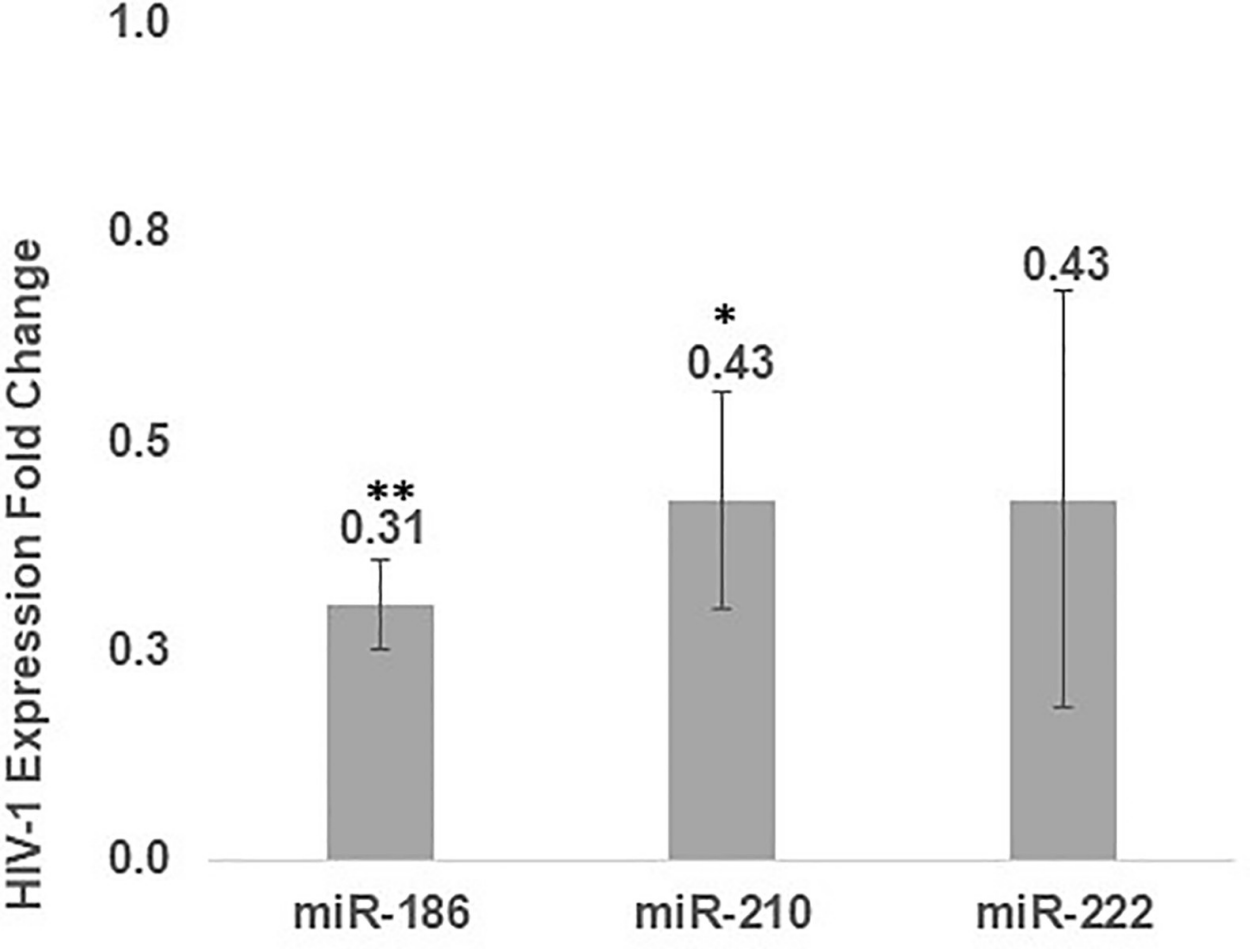
miRNAs-186, 210 and 222 affect HIV-1 replication. HeLa MAGI-CCR5 cells were transfected with the indicated miRNAs. Twenty-four hours later, the cells were infected with the HIV-1 virus. HIV-1 mRNA expression fold change was monitored by quantifying HIV-1 copies in the culture supernatants using RT-qPCR twenty-four hours post viral infection. Values are expressed as Expression Fold Change following miRNA over-expression compared to a control plasmid over-expression. The paired Student t-test was used for statistical analysis (n = 2, * p<0.05, ** p <0.005).

Finally, to establish the functional involvement of miR-186, 210 and 222 in HIV-1 replication, we transfected HeLa MAGI-CCR5 cells with miR-186, 210 or 222 twenty-four hours prior to HIV-1 infection. HIV-1 expression was determined by RT-qPCR of the HIV-1 in the supernatant 24 hours PI (S2 Table). A decrease in HIV-1 expression was detected with all three miRNAs tested, as shown in Fig 5.

Overall, we conclude that miR-186 and miR-210 alter the expression of HIV-related target mRNAs, and may play significant roles in viral replication. Additionally, miR-222 shows a similar phenotypic trend, though without statistically significance.

## Discussion

HIV-1 infection has been shown to promote the expression of various small RNA molecules that originate from both viral and human genomes, and that may affect the virus and host organism[2,32–35]. The involvement of these small RNA molecules in the pathogenesis of HIV-1 infection may be important for viral detection, as well as for gaining new insights regarding the infection process. In this work, we explored the expression profile of one type of small RNA–miRNA–during HIV-1 infection.

Here we studied the effect of human miRNA on its own mRNA expression using: small RNA deep sequencing data in SupT1 cells, previously published TLDA data, bioinformatics analysis and RT-qPCR experiments. We suggest that HIV-1 interferes with the human response to the infection, and alters levels of host miRNAs; this results in up/down-regulation of mRNAs, and potentially affects downstream protein levels related to key pathways (for example of complex pathway effects see [36]).

After infecting Supt-T1 cells for eight days, we identified and validated the altered expression of 12 miRNAs that we hypothesized to have important roles in the HIV-1 infection process. Bioinformatics analysis was applied to evaluate the potential target genes of these miRNAs, and revealed that miR-186, 210 and 222 were most up-regulated and were predicted to have 3 mutual human target genes–Dicer1, HRB and HIV-EP2.

Dicer is a type III cytoplasmic endoribonuclease that participates in the maturation of miRNAs[37]. Interestingly, Dicer was shown to affect viral RNA[38], but is also affected by HIV-1 infection. A recent study showed that Dicer alters virus-derived small RNA 1 (vsRNA1) generation, which in turn represses viral translation and IRES (internel ribosomal entry site) activity in infected cells[39]. Dicer was also shown to induce TAR element processing, yielding a viral miRNA involved in viral LTR chromatin remodeling[40]. Alternatively, Dicer was shown to be suppressed in HIV-1 infected macrophages via the viral gene Vpr[41], and the HIV-1 transcription factor Tat was reported to reduce Dicer activity[42]. As expected, Dicer reduction induces a decrease in miRNA expression[41,42].

**Fig 6:**
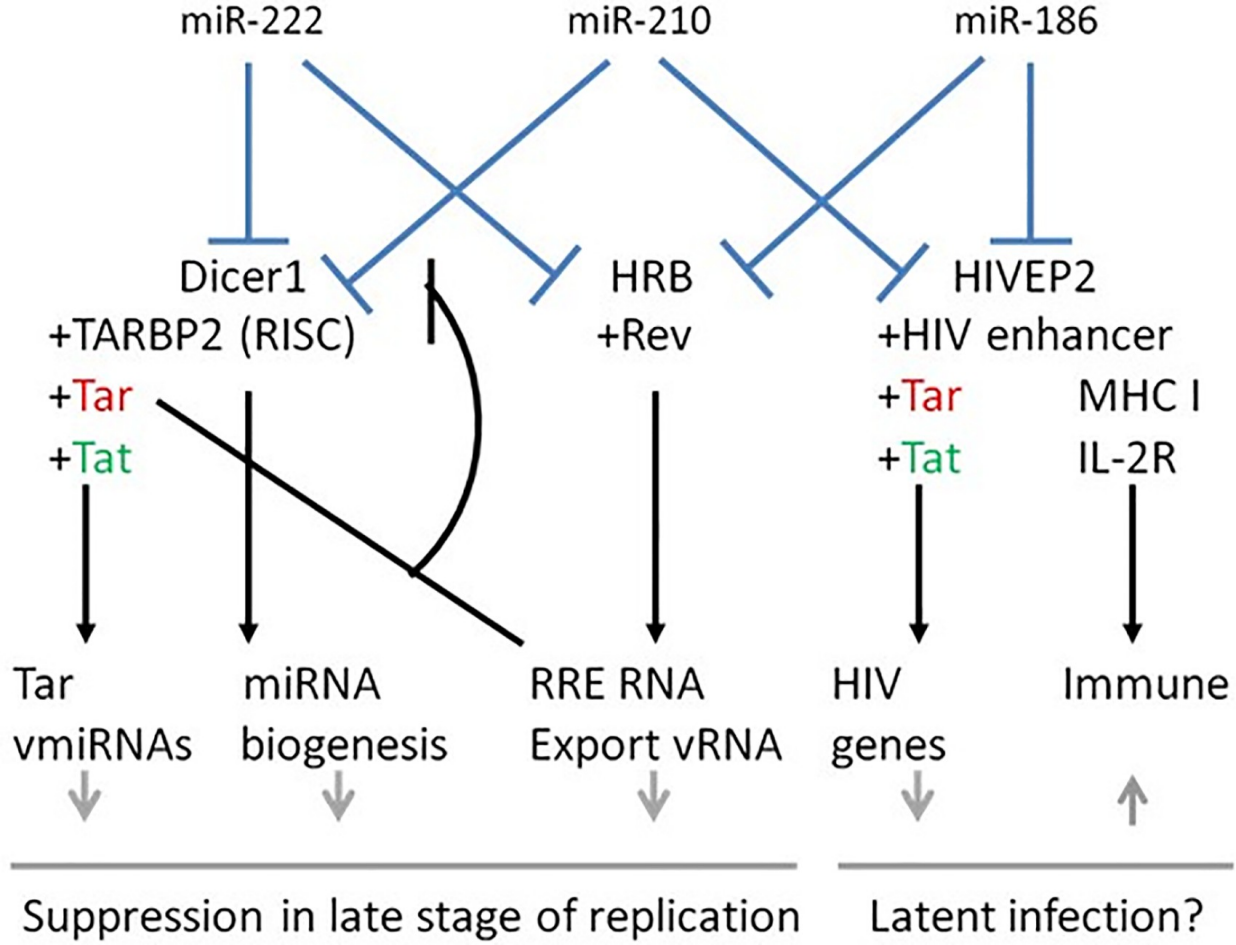
miRNAs-186, 210 and 222, their target genes and their potentially affected downstream pathways. A schematic diagram representing the possible interactions affecting cellular pathways.

Here, we demonstrated that upon HIV-1 infection, miR-222 expression was markedly increased in SupT1 cells and, consequently, Dicer and HRB mRNA expression decreased. The expression of miR-222 was previously shown in Jurkat cells [43]. The involvement of Dicer in the suppression of HIV-1 replication via the processing of the TAR element is accompanied by a feedback inhibition loop (see Fig 6). This is because HIV-1 induces a competitive interaction between TAR and RRE RNA, which suppresses the activity of miRNA machinery by interfering with Dicer’s interaction to HIV-1 TAR RNA binding protein (TRBP2)[34]. HRB binds to HIV Rev and exports partly spliced, RRE-bound HIV RNA from the nucleus periphery to the cytoplasm[44,45]. Therefore, we hypothesized that the reduced levels of HRB, following the up-regulation of miR-222, most likely induce a decrease in cytoplasmic RRE RNA. The Dicer-TRBP2 interaction is stabilized, resulting in the continuous processing of TAR into viral miRNAs (as demonstrated by Klase et al[40].). This in turn reduces LTR expression and enhances viral latency[40]. Our HIV-1 replication assay in MAGI-CCR5 cells supports this notion, as we detected a decrease in HIV-1 levels following miR-222 over-expression. Indeed, one study found that miR-222 expression is significantly induced in HIV-1-infected Jurkat T cells[46]. The authors of that study also showed the involvement of Tat protein in enhancing the transcriptional activity of NFkB on the miR-222 promoter, and the effect of the latter on down-regulating CD4, which is the key receptor for HIV-1.

In the current study, Dicer was also down-regulated by miR-210, which reduced expression levels of HIV-EP2 as well. HIV-EP2 is a large, zinc-finger containing transcription factor that plays a critical role in cell growth[47], signal transduction, apoptosis and differentiation regulation in T-cell development[44,45]. According to our analysis, HIV-EP2 down-regulation was induced by both miR-210 and miR-186. These findings correspond to the decrease in cellular HIV-EP2 levels, consequent to suppression in viral gene expression. We suspect that a similar effect occurs when HIV-EP2 interacts with IL-2R, as the IL-2/IL-2R regulatory determinant of T cell reactivity is known to be impaired during HIV-1 infection[48, 49, 50,51]. miR-210 was previously shown to be up-regulated in PBMCs and the Jurkat cell line, but down-regulated in CD8+ T cells of HIV-1 infected individuals[52,53]. Both miR-210 and 222 were associated with several inflammatory markers, suggesting they might play a role in regulating inflammation processes.

Based on our analysis, HRB and HIV-EP2 are potential targets of miR-186. Interestingly, mir-186 was shown to be significantly up-regulated (along with 10 other miRNAs) in subcutaneous adipose tissue of HIV-1 patients undergoing cART therapy[54]. The authors postulated that this up-regulation might contribute to the pathogenesis of HIV-associated lipodystrophy, by increasing cell turnover or promoting apoptosis. Our data regarding the involvement of miR-186 in HRB and HIV-EP2 regulation should be examined in depth, as it may shed light on the mechanisms that underlie the attenuated control of cellular-viral interactions. We note that the limitations of this study are that the experiments were conducted in a different cell line per experiment and miRNAs in infections assays were over expressed before viral infection.

Finally, a list of selected miRNAs was predicted to influence the human cell cycle and immune response pathways. MiRNA-186, 210 and 222 were suggested to be involved in a novel regulatory network of human miRNAs and target genes that influence HIV-1 latency. We demonstrated that these three miRNAs directly regulate the human genes Dicer1, HRB and HIV-EP2, thus filling key roles during the late stages of HIV-1 replication: in viral gene expression, in viral RNA transfer from the nucleus to the cytoplasm, and in the human and viral related miRNA biogenesis pathway (see Fig 6). The details of this regulation should be further studied with the goal of elucidating the cellular and viral interactions during HIV-1 replication.

## Supporting information

S1 Table Word file. (DOCX)

S2 Table Excel file. (XLSX)
