# Supplementary material for: HIV-1 infection increases microRNAs that inhibit Dicer1, HRB and HIV-EP2, thereby reducing viral replication
Source: PLoS One. 2019 Jan 25;14(1):e0211111. doi: 10.1371/journal.pone.0211111 (PMC6347224; doi:10.1371/journal.pone.0211111)
Supplement: S1 Table — (DOCX) [file pone.0211111.s001.docx]

Table S1. The constructs of 3'UTR for the luciferase reporter assay

| 3'UTR | Length (bp) | miRNA binding sites | Free energy [kcal/mol] | Primers sequences for 3'UTR constructs | Primers sequences for SDM |
| --- | --- | --- | --- | --- | --- |
| HRB | 420 | miR-222 | -91.50 | XhoI_HRB_222F: **acactcgag**tactggaaagtaacagagtcaa  NotI_HRB_222R: **aaggtcaagcggcc**gctgcaaagaaattagtaggat | mut_222 HRB F: gtaaagcatttaaatacctcttgtcagatttacc  mut_222 HRB R: ggtaaatctgacaagaggtatttaaatgctttac |
|  |  | miR-186 |  |  | mut_186 HRB F: ctatttatacatgcctaaataggcttaaaatgtagagggatacc  mut_186 HRB R: ggtatccctctacattttaagcctatttaggcatgtataaatag |
| Dicer1 | 363 | miR-222 | -68.30 | XhoI_dicer_222F: **acactcgag**attccttatgatgattgtgtgc  NotI_dicer_222R: **aaggtcaagcggcc**gcaatctgtttcatactttcacaa | mut_222 dicer1 F: cttctgttctgttcaaaccgcacagataagcatt  mut_222 dicer1 R: aatgcttatctgtgcggtttgaacagaacagaag |
|  |  | miR-210 |  |  | mut_210 dicer1 F: atgtgtcatatactctagaaacttaaataggtca  mut_210 dicer1 R: Tgacctatttaagtttctagagtatatgacacat |
| HIV-EP2 | 283 | miR-186 | -51.70 | XhoI_HIVEP_186F: **acactcgag**taagttacagtagtttgctatta  NotI_HIVEP_186R: **aaggtcaagcggcc**gctttacatcatttagatccaa | mut_186 HIV-EP2 F: gatgtttgtgcctttgcggtactttgcttatattc  mut_186 HIV-EP2 R: gaatataagcaaagtaccgcaaaggcacaaacatc |
|  | 268 | miR-210 | -65.10 | XhoI_HIVEP_210F: **acactcgag**tcacactcctggctatctca  NotI_HIVEP_210R: **aaggtcaagcggcc**gctacaaacttggtttcacagaat | mut_210 HIV-EP2 F:catgtgcacaggcacttgaatgtgtgcacgcaca  mut_210 HIV-EP2 R: tgtgcgtgcacacattcaagtgcctgtgcacatg |
